# Service Quality and Customer Satisfaction in Pharmaceutical Logistics: An Analysis Based on Kano Model and Importance-Satisfaction Model

**DOI:** 10.3390/ijerph16214091

**Published:** 2019-10-24

**Authors:** Mu-Chen Chen, Chia-Lin Hsu, Li-Hung Lee

**Affiliations:** 1 Department of Transportation and Logistics Management, National Chiao Tung University, Taipei 100, Taiwan; 2 Department of International Business Administration, Chinese Culture University, Taipei 11114, Taiwan; xjl6@ulive.pccu.edu.tw; 3 Department of Transportation and Logistics Management, National Chiao Tung University, Taipei 100, Taiwan; flliskimo@yahoo.com.tw

**Keywords:** pharmaceutical logistics, Kano model, service quality, customer satisfaction

## Abstract

The implementation of National Health Insurance in Taiwan has affected the medical industry by significantly depleting the supply chain’s profits. Service providers in the medical industry must meet the dual-service expectation of serving as medical manufacturers with upper reaches and as suppliers in the downstream marketing channel. As a result, service providers must anticipate customer requirements, offer new service items that align with customer demands and improve the quality of existing services. This study aims to examine consumer perspectives about service satisfaction in the domestic medical industry using Kano’s two-dimensional model. In addition, it employs the importance-satisfaction model to determine service items that need improvement. The empirical findings show that consumer perceptions about service quality attributes vary and thus, service items may be categorized differently in Kano’s model. Further, the reliability of service quality significantly affects customer satisfaction. Thus, service providers can gain a competitive edge and maintain their market position by offering high value added and critical quality attributes. Finally, analyzing customer attitudes toward new service items for indifference quality will help service providers determine effective tactics in a competitive market. In general, service providers should assign higher priority to items that customers consider in need of improvement.

## 1. Introduction

Since the implementation of National Health Insurance, the revenue and expenditure situation of the health insurance industry has been at a continual loss. Further, the Health Insurance Bureau has made several efforts to control the wastage of medical resources effectively and has adopted a series of policy measures to curb the growth of medical expenses, reduce problems related to healthcare finance and decrease drug prices. Measures, such as per-case payment, compensation-based payment, the total budget system and reasonable outpatient volume have significantly impacted medical institutions, resulting in a gradual decline in their revenue. Medical institutions initially demanded the use of the open-source method to expand business volume, although the total budget system and the mandate for reasonable outpatient volume were implemented. As a result, medical institutions were required to strengthen cost control using business management methods and reconsider the circulation of medical material.

In a hospital’s cost structure, personnel costs account for the highest proportion, followed by material cost and hospital purchases. From an operational perspective, medical institutions must (1) function in an open, fair and equitable bidding environment to obtain reasonable low-cost pharmaceutical materials; (2) reduce storage space to increase inventory turnover and expand space for medical services and (3) implement the automated collection of order lists to reduce in-hospital distribution frequency and zero inventory management through the systematic and effective management of inventory and order quantities. Hospitals have to consider ways to reduce expenses incurred for the various aspects of medical treatments. The industry explored, for example, the integration of logistics, which resulted in the outsourcing of pharmaceutical logistics for medical materials. The rapid and convenient distribution of logistics services is expected to promote the medical industry through a combination of logistics, information management and automated connection. This reduces unnecessary costs, creates additional profits, forms a general business model, decreases operating costs and meets the demand for limited diversified drugs and medical material, thereby enhancing the overall logistics performance of the medical industry supply chain.

The distribution channels of the pharmaceutical industry are relatively closed compared to those of other industries. Most pharmaceutical manufacturers engage with logistics or distribution companies that commission non-professional drugs, which offer poor logistics and distribution quality. Pharmaceutical logistics must meet the special needs of hospitals, clinics, pharmacies and homecare treatments, which include storage conditions as well as temperature and humidity control. In a distribution environment, it is increasingly important to maintain stability in medicine quality during inventory management and circulation.

Upstream domestic and foreign pharmaceutical manufacturers are constantly striving to survive this highly competitive industry with a large-scale medical supplies market. To reduce the overall inventory costs and accurately forecast market demand, manufacturers outsource logistics to professional third-party pharmaceutical logistics companies. In doing so, they seek cost, strategy and quality alternatives to reduce logistics costs, improve industry focus, enhance market competitiveness and eventually, earn higher profits.

Pharmaceutical logistics operators must meet the dual-service expectation of functioning as upstream pharmaceutical manufacturers and downstream marketers of medical products. To achieve the loyalty of customers and pharmaceutical companies and to gain market advantages, pharmaceutical logistics service providers must continually improve their service quality while accounting for input costs. However, once the quality improvement has reached a certain level, additional investments are unlikely to produce the same profits. Thus, it is important to examine how pharmaceutical logistics operators can better understand consumer needs over those of competitors and upstream pharmaceutical companies to provide services that satisfy consumer demand and improve service quality.

Improving service quality is a key competitive factor in a service-oriented business model. In their PZB (i.e., the abbreviation of Parasuraman, Zeithaml & Berry) model, Parasuraman et al. (1988) stated that quality is a measure of customer perception and is based on consumers’ subjective judgment [1]. That is, in addition to a company’s objective identification, service quality must be accounted for as the customers’ subjective identification to improve service level. Moreover, customers must be able to recognize the service quality so that service providers can determine if the improvements meet customer needs. In this customer-oriented era, convenience has gradually gained attention. The key focus of customer convenience is improving service standards, which, in turn, will be the basis for added customer support. Similarly, service convenience is an important aspect of logistics services.

Numerous industries and the service industry, in particular, are increasingly prioritizing customer satisfaction. Customer satisfaction is achieved by evoking feelings of loyalty and satisfaction in a customer. Service providers can gain customer attention by identifying customer interests from an empathy perspective and by offering products and services that resolve customer problems, thus creating a level of intimacy between the consumer and the service. Cognitive assessments conducted from a customer viewpoint suggest that convenient services are designed through continuous improvement aimed at achieving consumer attention and satisfaction.

To achieve consumer satisfaction, it is critical to first examine the customers’ opinions of service quality, understand related characteristics or dimensions to measure service and product quality, and strengthen quality factors that are of concern to customers. The most appropriate approach for achieving customer satisfaction is improving service quality. In addition to optimizing the use of limited resources, promptly responding to changing customer needs is critical to improve service quality and achieve customer satisfaction. This also plays an important role in enhancing industry competitiveness.

Adopting a narrow view of quality facets is unlikely to translate into a true understanding of the consumers’ quality perception. A one-dimensional quality view of customer satisfaction with products and services considers service quality to be a linear factor. To address the cognitive shortcomings of the one-dimensional quality view, Kano proposes a two-dimension model that states that satisfaction is achieved when certain quality requirements have been met, whereas the failure to do so leads to dissatisfaction [2]. The two-dimensional quality model evaluates customer satisfaction and its source on the basis of five quality factors: performance, basic, excitement, indifference and reverse.

Using the domestic pharmaceutical logistics industry as an example and the hospital industry as the research object, this study examines service quality and consumer satisfaction in the pharmaceutical logistics industry using the PZB model and Berry et al.’s definition of service convenience [3]. Further, it explores the key factors motivating the pharmaceutical industry to outsource services to major logistics providers in the domestic pharmaceutical industry. A key finding is that customers are attracted to services whose quality is aligned with their requirements.

## 2. Literature Review and Hypothesis Development

### 2.1. Pharmaceutical Logistics

Pharmaceutical logistics include the operations and activities such as warehousing, inventory management and transportation for pharmaceuticals. Many pharmaceutical products and medical devices are temperature sensitive and they need to be in a temperature-controlled environment. According to MarketWatch’s report, pharmaceuticals cold-chain logistics is defined as “*an uninterrupted series of refrigerated supply chain activities including refrigerated storage and transportation from their production point to destination of consumption* [4].” In recent years, the healthcare service industry has paid much attention on implementing logistics and supply chain management practice and strategy [5]. Logistics management is one of the critical enablers for successful services in the healthcare industry. As people expect efficient healthcare services, excellent pharmaceutical logistics are essential [6]. Several previous studies have indicated that healthcare organizations can substantially benefit from successful logistics management [7]. However, Landry et al. indicated that a hospital spends a sizable budget on logistics operations and if healthcare professionals need to spend too much time on logistics operations, the delivery of care is influenced [7].

Based on a report of Allied Market Research, Katare and Sonpimple indicated that transportation of temperature-controlled pharmaceutical products and medical devices has expanded substantially in the healthcare logistics industry [8]. Another report by Grand View Research, Inc., revealed that the market of global pharmaceutical logistics is sizeable, amounting to USD (i.e., United States Dollar) 76.4 billion in 2018. Furthermore, the demand for pharmaceutical logistics has risen substantially due to the requirement of supply chain reliability and the need to reduce distribution costs [9]. As medical products have unusual demand and supply and they are quality sensitive, special knowledge is entailed in the transportation operation [10]. Kumar and Jha indicated that maintaining stable temperature during transportation can avoid damage to medical products, and careful transportation management is also needed to avoid theft and mismanagement [10]. Summarizing the abovementioned reports and previous research, the demand for and market of pharmaceutical logistics have been increasing, and temperature control and management are essential for maintaining the quality of pharmaceutical products and medical devices. By using the analytical network process (ANP), Kritchanchai et al. found that the strategies related to inventory management and information and technology management are most essential to enhancing healthcare logistics performance [5].

### 2.2. Service Quality, Service Convenience, and Customer Satisfaction

Anderson and Sullivan argued that service quality is a pre-requisite of customer satisfaction [11]. Customer satisfaction depends on the performance of products and services and whether this performance is consistent with consumer expectations. Satisfaction is generally defined as the feeling of happiness customers experience from using products or services and it differs from expected performance. Understanding consumer satisfaction with service quality expectations and perceptions can serve as a basis to improve service quality, increase consumer repurchase, and enhance consumers’ willingness to recommend the service to others.

Early research focuses on resources expended by consumers to use a purchased good or service. Etgar, for example, defined convenience as a reduction in the non-monetary cost attributes of goods [12]. Brown stated that convenience is the extent of time and effort invested by a consumer to use a service and not the classification of product characteristics or attributes [13]. Berry et al. highlighted the lack of a conceptual framework for service convenience in which time and energy are important aspects and accordingly, listed various types of service convenience [3]. Their service convenience model classified convenience into decision-making, access, transaction, benefit and post-benefit convenience. Decision-making convenience is the time and effort consumers spend on deciding how to obtain a desired service. Access convenience is the time and energy invested in accessing a service. Transaction convenience is the amount of time and energy needed to conduct a transaction. Benefit convenience is defined as the time and energy needed to experience the core features of the service. Finally, post-benefit convenience is determined by the time and energy a consumer must invest in contacting a company following service usage.

The amount of time and effort needed to obtain a desired service reduces when consumers can easily understand product information and trust the professional ability and attitude of the service staff. This highlights both service and decision-making convenience. However, if business hours are considered inconvenient to the customer (e.g., customer service staff is difficult to contact) or if service providers cannot meet customer demands, the extent of time and energy needed from a customer increase. Both service and access convenience are exemplified in this case. When service personnel promptly serve customers and complete service transactions on a timely basis, customers’ invested energy and time reduces, suggesting both service and transaction convenience. These findings are in line with the PZB model. This study focuses on post-benefit and access convenience, not decision-making, transaction, and benefit convenience proposed by the PZB model, to measure customer satisfaction and service quality in the pharmaceutical logistics industry.

### 2.3. Kano Model

As per the one-dimensional quality model, customer satisfaction is achieved when certain quality requirements are sufficiently met, whereas the failure to do so results in dissatisfaction. However, not all quality factors can be characterized from such a one-dimensional perspective. Although different from the traditional one-dimensional model, the two-dimensional quality model suggests that not all quality factors lead to satisfaction and some may even result in dissatisfaction. Kano’s model draws on the proposal by psychologist, Herzberg, to use the motivator-hygiene theory aimed at job satisfaction and Dr. Ishikawa’s concept of backward quality that focuses on quality improvements. Kano et al. renamed these to attractive quality and must-be quality in their two-dimensional quality model [2]. The model uses the abscissa to denote the quality elements and ordinates to represent the degree of customer satisfaction. Kano further classified quality into attractive, one-dimensional, must-be, indifferent quality, and reverse quality.

Customers are satisfied when services have attractive quality, a desired quality whose absence leads to dissatisfaction. Even a small number of related quality elements significantly improves customer satisfaction, which can be further enhanced if the customers were not expecting these elements. In the one-dimensional quality model, customer satisfaction is determined by the availability of quality factors, where the higher the degree of quality, the greater the customer satisfaction. This suggests a linear relationship between customer satisfaction and factor supply. For instance, consumer satisfaction is not affected by the availability of must-be quality factors. However, their absence could cause severe customer dissatisfaction. Kano also defined quality factors from the viewpoint of expected quality, that is, consumers expect quality factors, and thus, they become basic characteristics of products and services. Notably, irrespective of the extent of quality factors provided, the curve is unlikely to exceed the horizontal axis. The indifferent quality factors do not influence customer satisfaction, while reverse quality factors could lead to customer dissatisfaction and thus, their absence is preferred. Kano’s two-dimensional quality model defined quality factors influencing customer satisfaction, and the findings can be used as a reference to develop and improve products and services [14]. In other words, companies must meet all basic quality requirements to achieve customer satisfaction and gain a competitive advantage to attract new customers [15].

### 2.4. Importance-Satisfaction (IS) Model

Yang examined the relationship between the importance assigned by customers to quality factors and customer satisfaction and found that both indicators contribute to an organization’s quality improvement and decision making [16]. When assessing the overall quality, customers respond to quality factors they consider important [16,17] and a company plays an important role in this evaluation. The projects with high quality but low satisfaction should be subject to quality improvement [16]. The importance-satisfaction (IS) model denotes the importance assigned to quality factors on the horizontal axis and the degree of satisfaction derived from quality factors on the vertical axis. Each quality factor is scored and the average or median of the importance assigned to quality factors lie in the center, creating four quadrants: excellent, to be improved, surplus, and careless. The quality elements located in the excellent quadrant are considered important by customers and lead to customer satisfaction. Thus, companies aiming to retain their customers must strive for high performance in this area. The elements in the to-be-improved area are critical but not as good as expected. Companies should work toward offering such elements or immediately improving existing factors. Customers do not prioritize quality elements in the surplus area. Thus, a company wanting to reduce costs can decrease the supply of such elements without negatively affecting customer satisfaction. Finally, the elements in the careless area are assigned low priority or considered unimportant. Companies can ignore such quality factors because they do not affect overall customer satisfaction.

### 2.5. Hypotheses Development

Kano’s model further divided quality into five dimensions on the basis of the importance assigned by consumers to quality factors and customer satisfaction: attractive, one-dimensional, must-be, indifferent and reverse quality [2]. The model defined customer satisfaction in line with these dimensions and the findings can serve as a reference for product or service development and improvement [14]. Some researchers (e.g., [18,19,20]) confirmed that customer perceptions about quality elements tend to differ and use Kano’s model to classify quality elements accordingly. In line with this approach, this study proposes the following hypothesis.

**Hypothesis** **1 (H1):** *The characteristics of quality elements for pharmaceutical logistics services differ in the Kano model.*

Businesses tend to focus on continuous improvements to their products and services. Numerous business firms employ customer satisfaction as an index to evaluate service performance as satisfied customers are more likely to repurchase and buy other products and services offered by the same firm [21]. Some studies confirmed that quality elements are determinants of customer satisfaction [19,22]. Kano et al. suggested that attractive and one-dimensional quality elements increase customer satisfaction, whereas the lack of one-dimensional and must-be quality elements results in customer dissatisfaction [2]. Matzler and Hinterhuber identified the elements that significantly influence customer satisfaction [14]. Accordingly, this study proposes the following hypothesis.

**Hypothesis** **2 (H2):** *Customer satisfaction is positively correlated with customers’ positive perceptions of attractive, one-dimensional, and must-be quality elements.*

Medical institutions have varying characteristics, and thus, their response to quality attributes of pharmaceutical logistics services tends to differ. Studies (e.g., [18,19,20]) confirmed that these varying characteristics result in quality elements being differently classified in the Kano model. This study, therefore, presents the following hypothesis.

**Hypothesis** **3 (H3):** *Medical institutions with different characteristics have varying views about the quality elements of medical logistics services.*

## 3. Methodology

### 3.1. Sample and Data Collection

The participants of this study are teaching and non-teaching hospitals that function under the Ministry of Health and Welfare, Executive Yuan. A total of 475 questionnaires were distributed for convenience sampling. Of these, 104 questionnaires were returned, indicating a response rate of 22%. Table 1 presents the sample’s demographics.

### 3.2. Questionnaire Design

The questionnaire was designed on the basis of the SERVQUAL (i.e., the abbreviation of service quality) scale developed by Parasuraman et al. [1] and the two dimensions of benefit and post-benefit convenience proposed by Berry et al. [3].

This study also employed Kano’s model to classify quality elements into attractive, one-dimensional, must-be, indifferent and reverse [2]. The questionnaire contains both functional (positive) and dysfunctional (negative) questions for each service element. The former is for customer responses to service elements and the latter documents responses to services that are not delivered. For example, “How would you feel if the delivery staff wore neat and tidy clothing?” is a functional question and “How would you feel if the delivery staff did not wear neat and tidy clothing?” is a dysfunctional question. The respondents’ replies were then subject to the quality classifications proposed in the Kano model.

Drawing on Matzler and Hinterhuber [14], the questionnaire offers five response options to classify service elements: “I like it,” “It must be that way,” “I am neutral,” “I don’t mind” and “I dislike it.” The service elements were classified into Kano element types on the basis of their scores. Referencing previous studies [18,19,20], the questionnaire also documents basic information such as information on companies and respondents. The options offer a reasonable level of clarity to the present investigation on pharmaceutical logistics services. Prior to formally administering the questionnaire, a pre-test was conducted with five users of logistics services for the healthcare cold chain. The questionnaire was modified according to the pre-test results.

The questionnaire comprises three parts: respondents’ demographics, functional and dysfunctional questions, and the importance of and satisfaction with pharmaceutical logistics services. A five-point Likert scale was used to measure the importance assigned to (1 is “very unimportant” and 5 is “very important”) and satisfaction derived from (1 is “very unsatisfied” and 5 “very satisfied”) the services offered by pharmaceutical logistics service providers.

## 4. Analysis and Results

### 4.1. Reliability and Validity Analysis

The reliability of each construct measured by the coefficient of Cronbach’s alpha exceeds 0.7, which is consistently high across all constructs. Cronbach’s alpha for all dimensions—tangibility, reliability, responsiveness, assurance, empathy, benefit convenience and post-benefit convenience—range between 0.781 and 0.968. This indicates that the constructs for these scales have high reliability. To gain insight on the relationships among these constructs, the two conditions of convergent validity and discriminant validity must be fitted.

The convergent validity test shows that the factor loadings for all measures of the underlying constructs exceed 0.5 (0.521–0.930). This confirms that the composite reliabilities of all constructs exceed the 0.7 cut-off value (0.78–0.86) recommended by Fornell and Larcker [23]. The average variance extracted from each construct exceeds 0.5 (0.53–0.67), indicating convergent validity [23]. In sum, the proposed constructs of the extended model are adequate as per the results of the convergent validity test.

Next, a discriminant validity test was conducted to assess the degree to which the constructs differ. If the items in a construct are more strongly correlated with each other than with the items measuring other constructs, the evaluation is considered to have discriminant validity. Table 2 presents the squared inter-correlations among the study constructs. In particular, it shows that the shared variance among the constructs does not exceed the square root of average variance explained. These results confirm discriminant validity.

### 4.2. Kano Model Results

#### 4.2.1. Kano Model Results for Respondents

Table 3 shows the 38 quality factors in this study. Specifically, four quality factors were classified under one-dimension quality, 23 quality factors were classified under must-be quality, 12 quality factors were classified under indifferent quality.

This study employed the Matzler and Hinterhuber’s [14] two-dimensional classification method to classify the items listed by the respondents under the five quality types. For example, the number of respondents who classify “cleanliness of distribution staff’s clothing” as a must-be quality is the highest (47.1%), followed by those who considered it a one-dimensional quality (26%), indifference quality (19.2%), attractive quality (6.7%) and invalid quality (1%). Therefore, “The clothing of the delivery staff is neat and tidy” is a must-be quality.

Evidently, opinions about quality factors differed by customer, and thus, this study adopted a statistical relative majority approach. The results reveal a significant difference in consumer opinions for two items: “There is a limit on the minimum order amount” and “Single-window customer service staff provides professional and satisfactory answers.” The classification of dimensional quality can, therefore, be confusing. Nevertheless, these difficulties do not extend to management and decision making [16].

The abovementioned results confirm that the variations in customers’ prioritization of quality factors in the pharmaceutical logistics services and that quality factors are differently classified in the Kano model. This supports Hypothesis 1 that the quality elements of pharmaceutical logistics services differ in quality characteristics classified in the Kano model.

#### 4.2.2. Categorization of Quality Elements Specified by Teaching and Non-Teaching Hospitals

This subsection focuses on understanding whether Kano’s two-dimensional quality categorization can be applied to quality the items mentioned by teaching and non-teaching hospitals (Table 4). There are significant differences in teaching and non-teaching hospitals’ views regarding five items: “Customer enquiries are answered within the promised period,” “Return and exchange processes are prompt and appropriate,” “Service personnel promptly addresses delivery errors,” “Out-of-stock orders are promptly processed” and “Customer complaints are immediately addressed and properly resolved.”

#### 4.2.3. Categorization of Quality Elements Listed by Logistics Service Providers

Table 5 lists quality elements mentioned by logistics service providers. This subsection determines if Kano’s two-dimensional quality classification can be applied to the quality items provided by service providers. The quality items related to pharmaceutical logistics companies most frequently contacted by customers are professional pharmaceutical logistics, general logistics, or both (Table 5). The results reveal significant differences among “Customer enquiries are answered within the promised period,” “Single-window customer service staff provides professional and satisfactory answers” and “Out-of-stock orders are quickly processed.” In sum, the respondents’ views of quality factors do not significantly differ on the basis of various hospital characteristics. Therefore, Hypothesis 3—that is, the quality of medical logistics services differs by the characteristics of medical institutions—is not supported.

### 4.3. Importance-Satisfaction Model (IS Model)

Customers assess the overall quality on the basis of quality factors they consider important [16,17]. The importance-satisfaction (IS) model can be further divided into four quadrants: excellent, to be improved, surplus and careless.

#### 4.3.1. Excellent

The quality elements that fall in the excellent quadrant are considered important by customers, and thus, service providers must focus on offering these features to achieve customer satisfaction. The following items are located in the excellent area: “Logistics company has good word-of-mouth, reputation, and popularity,” “Order content (i.e., items and quantities), bill of lading documents, and correctness of processing,” “Processes for batch numbers and validity period management are strictly implemented; e.g., drugs with a validity period of less than 6 months are not shipped,” “The rate and extent of damage received are disclosed/low,” “Logistics equipment and distribution vehicles meet the temperature requirements of drugs,” “Goods are promptly delivered to customers,” “Order (e.g., item and quantity) delivery accuracy rate,” “Customer enquiries are answered within the promised period,” “Return and exchange processes are prompt and appropriate,” “Urgent orders are accepted and processed with timely delivery,” “Single-window customer service staff provides professional and satisfactory answers,” “Service personnel (i.e., order and delivery personnel) promptly addresses delivery errors,” “Service staff (i.e., order and delivery staff) is kind and courteous,” “Customers are notified of product packaging changes in advance,” “Customer complaints are immediately addressed and properly resolved” and “Shipment of damaged goods or incorrect invoices and bills of voucher are promptly corrected.”

#### 4.3.2. To Be Improved

The to-be-improved area denotes the items important to customers but not as good as expected. The following elements fall under this category: “Customers are notified of shipment delays,” “Customers are notified of out-of-stock products” and “Out-of-stock orders are promptly processed.”

#### 4.3.3. Surplus

The items that fall within the surplus area are not prioritized by customers, and thus, companies attempting to reduce costs can decrease the supply of such items without negatively affecting customer satisfaction. The following items are located in the surplus area: “The clothing of delivery staff is neat and tidy,” “Logistics center has advanced physical equipment (e.g., warehouses, pickup systems, and shelves),” “Deliveries are completed every other day after receiving the order,” “Deliveries are made as per time specified by customers,” “Designated inventory location or shelf service are provided as per customer needs” and “Batch number and validity period requirements specified by the customer are met.”

#### 4.3.4. Careless

Customers consider the elements that fall in the careless area unimportant, and thus, companies are not required to prioritize the quality of these elements. The following items are located in the careless area: “There is a limit on minimum order amounts,” “Customers are notified of item shipment one day prior,” “Service personnel (i.e., order and delivery personnel) have received professional training and a certain degree of understanding about drugs,” “Customized logistics processing and packaging services are available,” “There is no limit on order time,” “Customers can place online orders using the electronic platform/Online orders are accepted,” “Delivery services are available on weekends and holidays,” “Customers can lodge complaints through their customer complaints channel,” “Customers receive order processing status online,” “Information on materials related to healthcare medicine and procurement advice are provided,” “Services such as medical waste recycling, waste disposal, and autoclaving are available” and “Services for escrow inventory are provided.”

### 4.4. Summary of Two-Dimensional Quality Elements

Table 6 summarizes the quality elements for pharmaceutical logistics services using Kano’s two-dimensional quality model. The table includes the importance and satisfaction scores as well as attribute classifications under the Kano and IS models. A noteworthy observation is that the quality elements for pharmaceutical logistics services do not report varying attribute classifications under the Kano model.

## 5. Discussion

This study examined medical institutions’ views of service quality for the domestic pharmaceutical logistics industry using Kano’s two-dimensional model and the IS model. The objective was to determine the service quality elements that attract customers to pharmaceutical logistics services and that meet medical institutions’ needs. The hypotheses test results indicate that pharmaceutical logistics services have varying quality characteristics. The two-dimensional quality classification must be positively correlated with satisfaction derived from the quality factor. Further, there is no significant difference in the classification of quality elements listed by medical institutions with varying characteristics.

### 5.1. Kano Model Results

The results of Kano’s two-dimensional quality highlight that of the 38 quality items, none are classified under attractive or reverse quality, four items are categorized under one-dimensional quality, 24 items fall under must-be quality, and 12 are considered non-differential qualities. A majority of the quality items are classified as must-be quality, indifference quality, or both.

Most of the items classified as must-be quality are services currently offered by the pharmaceutical logistics industry. For example, the following items are deemed basic characteristics of pharmaceutical logistics services: “Items of order content (i.e., items and quantities), bill of lading documents, and correctness of processing,” “Processes for batch numbers and validity period management are strictly implemented; e.g., drugs with a validity period of less than 6 months are not shipped,” “Logistics equipment and distribution vehicles meet the temperature requirements of drugs,” ‘Goods are delivered to customers on time,” “Order (i.e., item and quantity) delivery accuracy rate” and “Customer complaints are immediately addressed and properly resolved,” These factors do not enhance customer satisfaction. However, their absence could lead to customer dissatisfaction [2]. Both the tangible and reliable items of pharmaceutical logistics services are classified under must-be quality. In other words, service personnel and equipment for pharmaceutical logistics should aim to provide promised services correctly and reliably to meet the basic requirements of medical institutions.

The following items are classified as indifferent elements: “Customers are notified of item shipment one day prior,” “Service personnel (i.e., order and delivery personnel) have received professional training and a certain degree of understanding about drugs,” “Delivery services are available on weekends and holidays,” “Customers are provided order-processing status online,” “Services such as medical waste recycling, waste disposal, and autoclaving are available” and “Escrow inventory services are provided.” While the lack of these quality items does not affect customer satisfaction with pharmaceutical logistics providers, these factors may become attractive qualities. Thus, companies should consider providing such quality elements (e.g., “There is a limit on the minimum order amount”) as a strategic tool to attract customers in the future.

In sum, an important strategic implication offered by the findings of Kano’s two-dimensional quality model is that pharmaceutical logistics operators should provide attractive qualities to attract future customers and gain a competitive advantage. At the same time, these factors must satisfy the criteria of must-be quality and one-dimension quality so that service companies can gain a competitive advantage of their competitors.

#### IS Analysis Results

The IS model analysis categorizes the following items under the excellent area: “Customer complaints are immediately addressed and properly resolved,” “Urgent orders are accepted and processed with timely delivery” and “Processes for batch numbers and validity period management are strictly implemented; e.g., drugs with a validity period of less than 6 months are not shipped.” In other words, service providers in the pharmaceutical logistics industry must continuously and adequately provide these services to achieve customer satisfaction.

The following items are located in the to-be-improved area: “Out-of-stock orders are promptly processed,” “Customers are notified of delayed shipment” and “Customers are notified of out-of-stock products.” Service providers in the pharmaceutical logistics must immediately and aggressively improve these items to ensure customer satisfaction.

The following items fall in the surplus area: “Deliveries are completed every other day after receiving the order,” “Logistics center has advanced physical equipment (e.g., warehouses, pickup systems, and shelves)” and “Batch number and validity period requirements specified by the customer are met.” Customers do not prioritize these services, and the satisfaction obtained from the availability of these services is above average. Thus, companies attempting to reduce costs can direct their resources toward service items other than these without significantly impacting customer satisfaction.

Finally, the following items fall in the careless quadrant: “Delivery services are available on weekends and holidays,” “Customers are notified of item shipment one day prior,” “Customers are provided with order-processing status online,” “Customers can place orders online using the electronic platform/Online orders are accepted” and “Escrow inventory services are provided.” These items are less valued by customers, and thus, their contribution to customer satisfaction is limited. Therefore, companies do not need to spend much time and resources on these items and may even consider excluding these items to reduce costs.

### 5.2. Satisfaction Analysis for Different Service Providers

Table 7 shows that the following items fall in the excellent quadrant: “ Processes for batch numbers and validity period management are strictly implemented; e.g., drugs with a validity period of less than 6 months are not shipped,” “Single-window customer service staff provide professional and satisfactory answers,” “Deliveries are completed every other day after receiving the orders” and “Batch number and validity period requirements specified by the customer are met.” This finding indicates that customers prefer professional pharmaceutical logistics services. Further, the following items are located in the to-be-improved quadrant: “Customer enquiries are answered within the promised period,” “Return and exchange processes are prompt and appropriate” and “Customer complaints are immediately addressed and properly resolved.” Customers are satisfied with these items, but a small proportion believes they are not as good as expected. The item “Inventory location or shelf service is determined as per customer needs” is located in the surplus quadrant for professional pharmaceutical logistics services. However, this item is in the to-be-improved quadrant for general pharmaceutical logistics services. In other words, customers are satisfied with these services and at the same time, companies may consider excluding them to reduce costs. Similarly, “Deliveries are made as per time specified by customers” is located in the surplus area for general pharmaceutical logistics services, but it is in the excellent quadrant for professional pharmaceutical logistics services.

### 5.3. Research Limitations and Suggestions for Future Research

While this study was designed to be extended, certain areas remain that need to be addressed in future research. First, this study focuses on users of pharmaceutical logistics services in Taiwan using a questionnaire survey and thus, it does not account for customer needs in other countries. Researchers may consider surveying pharmaceutical logistics service providers in other countries to understand issues related to new services and to improve the generalizability of the results. Second, the questionnaire is mainly designed for downstream customers, such as medical institutions, and does not consider the views of upstream customers, including drug manufacturers. The research subjects are medical institutions, including pharmacies, clinics and homecare. Thus, there is scope to further explore the quality of services provided in the domestic pharmaceutical logistics industry. Finally, this study adopts a closed questionnaire method to explore customer perceptions of service quality in the pharmaceutical logistics industry. The omission of certain factors may have weakened the explanatory power. Future research could conduct theoretical analyses and in-depth interviews to examine other factors affecting service quality to supplement and revise the scale of pharmaceutical logistics services.

## 6. Implications

### 6.1. Managerial Implications of Kano’s Two-Dimensional Quality Model

This study adopted Kano’s two-dimensional quality model and a questionnaire survey to examine customers’ perceptions about service items and accordingly, classify the service items under various quality factors. These findings can serve as a reference for service providers in the pharmaceutical logistics industry working toward improving their services.

#### 6.1.1. Offer all Elements Classified under Must-Be Quality

Service items generally classified as must-be quality are those considered basic services by customers. Therefore, while the presence of these may not increase customer satisfaction, their absence could result in customer dissatisfaction [2]. The present empirical results suggest that tangibility and reliability are key attributes contributing to must-be quality. In the present context, this means medical institutions will demand high service standards, such as compliance with rules and regulations for the circulation of medicines. Certain related items that are classified as must-be quality are “The clothing of delivery staff is neat and tidy,” “Logistics equipment and distribution vehicles meet the temperature requirement of drugs,” “Order content (i.e., items and quantities), bill of lading documents, and correctness of processing” and “Processes for batch numbers and validity period management are strictly implemented, e.g., drugs with a validity period of less than 6 months are not shipped.” This means wearing clean clothing during drug circulation, satisfying drug temperature requirements and strictly monitoring batch numbers and expiration dates are some of the quality control measures that should be adopted by the pharmaceutical logistics service industry.

#### 6.1.2. Strengthen Provisions of One-Dimensional Quality Elements

The availability of one-dimensional quality items positively contributes to customer satisfaction. A higher number of one-dimensional quality items will result in greater customer satisfaction, whereas the lack of these items will lead to customer dissatisfaction [2]. For example, “Urgent orders are accepted and processed with timely delivery” is considered an essential service. Given the generally urgent demand for medicines, pharmaceutical logistics service providers must ensure prompt delivery, improve the efficiency of logistical operations, and decrease service delivery time, all of which could contribute to higher customer satisfaction.

#### 6.1.3. Focus on Providing Services and Products with Attractive Quality

The need to provide products and services with attractive qualities could motivate the industry to actively innovate. Offering products and services with sufficient attractive quality are likely to enhance customer satisfaction [17]. However, the empirical results of this study highlight that no item is classified under attractive quality, indicating that the current medical logistics industry is not highly innovative. Therefore, if the pharmaceutical logistics industry is committed to providing services with attractive qualities, it must explore reverse logistics services that are based on customer needs and differentiated strategies to gain a competitive advantage over competitors.

## 7. Conclusions

This study examined critical quality factors that influence customer satisfaction using Kano’s two-dimensional model and the IS model. In doing so, it also shed light on factors that are inadequate and need strengthening. The empirical findings offer key managerial implications for service providers of the pharmaceuticals logistics industry to improve their services by, for example, exploring innovative service content that accounts for customer needs. First, most service items cited by medical institutions can be classified under one-dimensional or must-be quality. Thus, medical logistics service providers should strengthen the integrity of their core services. Next, the items such as “Customers are notified of delayed shipment” and “Out-of-stock orders are promptly processed” fall in the to-be-improved quadrant. This means customers consider these aspects important, but they do not meet customer expectations. Companies should focus on improving the quality of such services. Finally, in addition to offering services in prestigious areas, professional pharmaceutical logistics offer better and different services than those provided by general logistics service providers. For example, professional pharmaceutical logistics operators ensure the strict enforcement of batch and expiration management processes, have well-trained customer service staff, offer prompt delivery once the order is received and deliver products and services as per the customers’ batch number and drug validity period. In other words, professional logistics service providers differ from general ones in management and timeliness. When the quality of services is high, customers tend to classify the items under one-dimensional quality or must-be quality because customers generally take these items for granted. By contrast, items that unavailable are classified as attractive or indifference quality. However, no item has been classified as attractive quality and the market segmentation is likely to differ by attributes prioritized by customers.

## Figures and Tables

**Table 1:** Demographic statistics.

Characteristics		Frequency	Percentage (%)
Area	North	30	28.85
	Middle	33	31.73
	South	35	33.65
	Other	6	5.77
Gender	Male	31	29.81
	Female	73	70.19
Age	≥24	3	2.88
	25–34	41	39.42
	35–49	47	45.19
	50–64	11	10.58
	≤65	2	1.92
Education level	High school or below	8	7.69
	College	92	88.46
	Graduate school or above	4	3.85
Seniority	≤3	19	18.27
	4–6	23	22.12
	7–10	28	26.92
	11–15	13	12.50
	≥16	21	20.19
Using pharmaceutical logistics time	≤3	34	32.69
	4–6	29	27.88
	7–10	25	24.04
	11–15	9	8.65
	≥16	7	6.73
The most frequently used pharmaceutical logistics type	Professional pharmaceutical logistics	69	66.35
	General logistics	17	16.34
	Both	18	17.31

**Table 2:** Discriminant validity analysis.

	Tan	Rel	Res	Ass	Emp	BC	PBC
Tan	0.692						
Rel	0.385	0.856					
Res	0.395	0.333	0.683				
Ass	0.405	0.276	0.522	0.767			
Emp	0.395	0.478	0.625	0.453	0.667		
BC	0.277	0.396	0.450	0.270	0.534	0.710	
PBC	0.388	0.276	0.602	0.388	0.547	0.541	0.695

Notes: Tan: Tangibility; Rel: Reliability; Res: Responsiveness; Ass: Assurance; Emp: Empathy; BC: Benefit convenience; PBC: Post-benefit convenience; All correlations are significant at the 0.05 level. The diagonals represent the square root of average variance extracted.

**Table 3:** Categorization of quality elements listed by respondents (%).

	Elements	AQ	MBQ	ODQ	IDQ	RQ	IVQ	Categorization of Quality Elements
1	The clothing of delivery staff is neat and tidy	6.7	**47.1**	26	19.2	0	1	MBQ
2	Logistics company has good word-of-mouth, reputation, and popularity	9.6	**40.4**	32.7	16.3	1	0	MBQ
3	Logistics center has advanced physical equipment (e.g., warehouses, pickup systems, shelves)	11.5	**40.4**	16.3	30.8	1	0	MBQ
4	Processes including order content (items and quantities) bill of lading documents are correctly executed	3.8	**47.1**	39.4	8.7	0	1	MBQ
5	Processes for batch numbers and validity period management are strictly implemented; i.e., drugs with a validity period of less than 6 months are not shipped	1.0	**48.1**	46.2	2.9	1.9	0	MBQ
6	The rate and extent of damage received are disclosed/low	1.0	**50.0**	43.3	4.8	1	0	MBQ
7	Logistics equipment and distribution vehicles meet the temperature requirements of drugs	1.9	**51.0**	37.5	8.7	1	0	MBQ
8	Goods are delivered on time to customers	1.0	**59.6**	34.6	3.8	1	0	MBQ
9	The order (i.e., item and quantity) delivery rate is accurate	0.0	**58.7**	34.6	6.7	0	0	MBQ
10	Customer enquiries are answered within the promised period	5.8	**43.3**	38.5	11.5	1	0	MBQ
11	Return and exchange processes are prompt and appropriate	4.8	**47.1**	39.4	5.8	1	1.9	MBQ
12	Urgent orders are accepted and processed with timely delivery	5.8	40.4	**43.3**	10.6	0	0	ODQ
13	There is a limit on the minimum order amount	19.2	23.1	**26.9**	**26.9**	1	2.9	ODQ/**IDQ**
14	Customers are notified of product shipment one day prior	19.2	12.5	10.6	**51.9**	2.9	2.9	**IDQ**
15	Single-window customer service staff provides professional and satisfactory answers	17.3	**29.8**	21.2	**29.8**	1	1	MBQ/**IDQ**
16	Deliveries are completed every other day after receiving the order	14.4	27.9	**34.6**	21.2	1.9	0	ODQ
17	Service personnel (i.e., order and delivery personnel) quickly address delivery errors	4.8	**54.8**	32.7	5.8	1.9	0	MBQ
18	Customers are notified of delayed shipment	3.8	**51.9**	36.5	3.8	1.9	1.9	MBQ
19	Service personnel (i.e., order and delivery personnel) have professional training and certain degree of understanding about drugs	25	26	8.7	**38.5**	1.9	0	**IDQ**
20	Service staff (i.e., order and delivery staff) are kind and courteous	5.8	**51.9**	35.6	5.8	1	0	MBQ
21	Customized logistics processing and packaging services are available	20.2	26.9	15.4	**35.6**	1	1	**IDQ**
22	Deliveries are made as per time specified by customers	12.5	**38.5**	33.7	13.5	1.9	0	MBQ
23	Customers are notified of out-of-stock products	4.8	**49.0**	34.6	6.7	2.9	1.9	MBQ
24	There is no limit on order time	18.3	18.3	24.0	**36.5**	2.9	0	IDQ
25	Customers can place online orders using the electronic platform/Online orders are accepted	22.1	17.3	10.6	**47.1**	1.9	1	IDQ
26	Delivery services are available on weekends and holidays	19.2	16.3	13.5	**47.1**	2.9	1	IDQ
27	There are channels for customer complaints	2.9	**53.8**	26	14.4	2.9	0	MBQ
28	Inventory location or shelf service is designated as per customer needs	9.6	**36.5**	30.8	21.2	1	1	MBQ
29	Information technology (RFID and barcode) offers information on drug history and temperature control	16.3	24	**37.5**	28.8	2.9	0	ODQ
30	Customers are notified of product packaging changes in advance	1.9	**56.7**	29.8	9.6	1.9	0	MBQ
31	Batch number and validity period requirements specified by the customer are met	5.8	**39.4**	25	28.8	1	0	MBQ
32	Customers receive order-processing status online	21.2	26	18.3	**32.7**	1	1	IDQ
33	Out-of-stock orders are promptly processed	1.9	**50.0**	39.4	4.8	1	2.9	MBQ
34	Customer complaints are immediately addressed and resolved	1.9	**55.8**	32.7	5.8	1.9	1.9	MBQ
35	Shipment of damaged goods or incorrect invoices and bills of voucher are promptly corrected	1.9	**51.9**	36.5	6.7	1	1.9	MBQ
36	Information on materials related to healthcare medicines and procurement advice are provided	23.1	21.2	17.3	**36.5**	1	1	IDQ
37	Services such as medical waste recycling, waste disposal, and autoclaving are available	23.1	18.3	16.3	**39.4**	1.9	1	IDQ
38	Escrow inventory services are provided	16.3	10.6	10.6	**56.7**	3.8	1.9	IDQ

Notes: AQ, attractive quality; MBQ, must-by quality; ODQ, one-dimension quality; IDQ, indifferent quality; RQ, reverse quality; IVQ, invalid quality. The bold stands for the majority.

**Table 4:** Categorization of quality elements by teaching hospital and non-teaching hospitals.

Items		Teaching	Non-Teaching	*p*-Value
1	The clothing of delivery staff is neat and tidy	MBQ	MBQ	-
2	Logistics company has good word-of-mouth, reputation, and popularity	ODQ	MBQ	-
3	Logistics center has advanced physical equipment (e.g., warehouses, pickup systems, shelves)	MBQ/IDQ	MBQ	-
4	Processes including order content (items and quantities) bill of lading documents are correctly executed	MBQ	MBQ/ODQ	-
5	Processes for batch numbers and validity period management are strictly implemented; i.e., drugs with a validity period of less than 6 months are not shipped	MBQ	MBQ	-
6	The rate and extent of damage received are disclosed/low	MBQ	ODQ	-
7	Logistics equipment and distribution vehicles meet the temperature requirements of drugs	MBQ	MBQ	-
8	Goods are delivered on time to customers	MBQ	MBQ	-
9	The order (i.e., item and quantity) delivery rate is accurate	MBQ	MBQ	-
10	Customer enquiries are answered within the promised period	ODQ	MBQ	*
11	Return and exchange processes are prompt and appropriate	ODQ	MBQ	*
12	Urgent orders are accepted and processed with timely delivery	ODQ	MBQ	-
13	There is a limit on the minimum order amount	ODQ	IDQ	-
14	Customers are notified of product shipment one day prior	IDQ	IDQ	-
15	Single-window customer service staff provides professional and satisfactory answers	ODQ/IDQ	MBQ	-
16	Deliveries are completed every other day after receiving the order	ODQ	ODQ	-
17	Service personnel (i.e., order and delivery personnel) quickly address delivery errors	ODQ	MBQ	*
18	Customers are notified of delayed shipment	ODQ	MBQ	-
19	Service personnel (i.e., order and delivery personnel) have professional training and certain degree of understanding about drugs	IDQ	IDQ	-
20	Service staff (i.e., order and delivery staff) are kind and courteous	MBQ	MBQ	-
21	Customized logistics processing and packaging services are available	IDQ	IDQ	-
22	Deliveries are made as per time specified by customers	MBQ	MBQ	-
23	Customers are notified of out-of-stock products	MBQ	MBQ	-
24	There is no limit on order time	ODQ	IDQ	-
25	Customers can place online orders using the electronic platform/Online orders are accepted	IDQ	IDQ	-
26	Delivery services are available on weekends and holidays	IDQ	IDQ	-
27	There are channels for customer complaints	MBQ	MBQ	-
28	Inventory location or shelf service is designated as per customer needs	MBQ	MBQ	-
29	Information technology (RFID and barcode) offers information on drug history and temperature control	IDQ	ODQ	-
30	Customers are notified of product packaging changes in advance	MBQ	MBQ	-
31	Batch number and validity period requirements specified by the customer are met	MBQ	MBQ	-
32	Customers receive order-processing status online	IDQ	IDQ	-
33	Out-of-stock orders are promptly processed	ODQ	MBQ	*
34	Customer complaints are immediately addressed and resolved	MBQ	MBQ	*
35	Shipment of damaged goods or incorrect invoices and bills of voucher are promptly corrected	MBQ/ODQ	MBQ	-
36	Information on materials related to healthcare medicines and procurement advice are provided	IDQ	IDQ	-
37	Services such as medical waste recycling, waste disposal, and autoclaving are available	IDQ	IDQ	-
38	Escrow inventory services are provided	IDQ	IDQ	-

Note: * *p* < 0.1; - means non-significant.

**Table 5:** Categorization of quality elements by logistics service providers.

Items		Professional	General	Both	*p*-Value
1	The clothing of delivery staff is neat and tidy	MBQ	MBQ	MBQ	-
2	Logistics company has good word-of-mouth, reputation, and popularity	MBQ	MBQ	MBQ	-
3	Logistics center has advanced physical equipment (e.g., warehouses, pickup systems, shelves)	MBQ	MBQ/IDQ	MBQ	-
4	Processes including order content (items and quantities) bill of lading documents are correctly executed	MBQ	MBQ	MBQ	-
5	Processes for batch numbers and validity period management are strictly implemented; i.e., drugs with a validity period of less than 6 months are not shipped	MBQ	MBQ	ODQ	-
6	The rate and extent of damage received are disclosed/low	ODQ	MBQ	MBQ	-
7	Logistics equipment and distribution vehicles meet the temperature requirements of drugs	MBQ	MBQ	MBQ	-
8	Goods are delivered on time to customers	MBQ	MBQ	MBQ	-
9	The order (i.e., item and quantity) delivery rate is accurate	MBQ	MBQ	ODQ	-
10	Customer enquiries are answered within the promised period	MBQ	MBQ	ODQ	*
11	Return and exchange processes are prompt and appropriate	MBQ	MBQ	ODQ	-
12	Urgent orders are accepted and processed with timely delivery	MBQ	MBQ/ODQ	ODQ	-
13	There is a limit on the minimum order amount	IDQ	ODQ	MBQ/ODQ/IDQ	-
14	Customers are notified of product shipment one day prior	IDQ	ODQ	IDQ	-
15	Single-window customer service staff provides professional and satisfactory answers	MBQ	IDQ	ODQ/IDQ	*
16	Deliveries are completed every other day after receiving the order	ODQ	IDQ	ODQ	-
17	Service personnel (i.e., order and delivery personnel) quickly address delivery errors	MBQ	MBQ	MBQ	-
18	Customers are notified of delayed shipment	MBQ	MBQ	MBQ	-
19	Service personnel (i.e., order and delivery personnel) have professional training and certain degree of understanding about drugs	IDQ	IDQ	IDQ	-
20	Service staff (i.e., order and delivery staff) are kind and courteous	MBQ	MBQ	MBQ	-
21	Customized logistics processing and packaging services are available	IDQ	MBQ	MBQ	-
22	Deliveries are made as per time specified by customers	ODQ	MBQ	MBQ	-
23	Customers are notified of out-of-stock products	MBQ	MBQ	MBQ	-
24	There is no limit on order time	IDQ	IDQ	ODQ	-
25	Customers can place online orders using the electronic platform/Online orders are accepted	IDQ	IDQ	IDQ	-
26	Delivery services are available on weekends and holidays	IDQ	IDQ	IDQ	-
27	There are channels for customer complaints	MBQ	MBQ	MBQ	-
28	Inventory location or shelf service is designated as per customer needs	MBQ	MBQ/ODQ	MBQ	-
29	Information technology (RFID and barcode) offers information on drug history and temperature control	ODQ	IDQ	ODQ	-
30	Customers are notified of product packaging changes in advance	MBQ	MBQ	MBQ	-
31	Batch number and validity period requirements specified by the customer are met	MBQ	MBQ	MBQ	-
32	Customers receive order-processing status online	IDQ	IDQ	MBQ/AQ	-
33	Out-of-stock orders are promptly processed	MBQ	MBQ	ODQ	*
34	Customer complaints are immediately addressed and resolved	MBQ	MBQ	MBQ	-
35	Shipment of damaged goods or incorrect invoices and bills of voucher are promptly corrected	MBQ	MBQ	MBQ	-
36	Information on materials related to healthcare medicines and procurement advice are provided	IDQ	MBQ/AQ	IDQ	-
37	Services such as medical waste recycling, waste disposal, and autoclaving are available	IDQ	IDQ	MBQ/ODQ/IDQ	-
38	Escrow inventory services are provided	IDQ	IDQ	IDQ	-

Note: * *p* < 0.1; - means non-significant.

**Table 6:** Summary of quality elements for pharmaceutical logistics services.

Items		Importance (Avg.)	Satisfaction (Avg.)	Kano’ Attribute Classification	IS Model
1	The clothing of delivery staff is neat and tidy	3.95	**3.94**	MBQ	Surplus
2	Logistics company has good word-of-mouth, reputation, and popularity	**4.12**	**4.06**	MBQ	Excellent
3	Logistics center has advanced physical equipment (e.g., warehouses, pickup systems, shelves)	3.93	**3.84**	MBQ	Surplus
4	Processes including order content (items and quantities) bill of lading documents are correctly executed	**4.40**	**4.14**	MBQ	Excellent
5	Processes for batch numbers and validity period management are strictly implemented; i.e., drugs with a validity period of less than 6 months are not shipped	**4.52**	**3.96**	MBQ	Excellent
6	The rate and extent of damage received are disclosed/low	**4.50**	**4.16**	MBQ	Excellent
7	Logistics equipment and distribution vehicles meet the temperature requirements of drugs	**4.41**	**4.12**	MBQ	Excellent
8	Goods are delivered on time to customers	**4.52**	**4.08**	MBQ	Excellent
9	The order (i.e., item and quantity) delivery rate is accurate	**4.56**	**4.18**	MBQ	Excellent
10	Customer enquiries are answered within the promised period	**4.26**	**3.86**	MBQ	Excellent
11	Return and exchange processes are prompt and appropriate	**4.35**	**3.72**	MBQ	Excellent
12	Urgent orders are accepted and processed with timely delivery	**4.40**	**4.00**	ODQ	Excellent
13	There is a limit on the minimum order amount	3.53	3.33	ODQ/IDQ	Care-free
14	Customers are notified of product shipment one day prior	3.25	3.28	IDQ	Care-free
15	Single-window customer service staff provides professional and satisfactory answers	**4.06**	**3.81**	MBQ/IDQ	Excellent
16	Deliveries are completed every other day after receiving the order	4.01	**3.89**	ODQ	Surplus
17	Service personnel (i.e., order and delivery personnel) quickly address delivery errors	**4.25**	**3.92**	MBQ	Excellent
18	Customers are notified of delayed shipment	**4.34**	3.41	MBQ	To be improved
19	Service personnel (i.e., order and delivery personnel) have professional training and certain degree of understanding about drugs	3.66	3.56	IDQ	Care-free
20	Service staff (i.e., order and delivery staff) are kind and courteous	**4.24**	**4.25**	MBQ	Excellent
21	Customized logistics processing and packaging services are available	3.51	3.48	IDQ	Care-free
22	Deliveries are made as per time specified by customers	3.99	**3.80**	MBQ	Surplus
23	Customers are notified of out-of-stock products	**4.36**	3.41	MBQ	To be improved
24	There is no limit on order time	3.72	3.43	IDQ	Care-free
25	Customers can place online orders using the electronic platform/Online orders are accepted	3.54	3.38	IDQ	Care-free
26	Delivery services are available on weekends and holidays	3.48	3.38	IDQ	Care-free
27	There are channels for customer complaints	3.96	3.65	MBQ	Care-free
28	Inventory location or shelf service is designated as per customer needs	3.93	**3.84**	MBQ	Surplus
29	Information technology (RFID and barcode) offers information on drug history and temperature control	3.81	3.47	ODQ	Care-free
30	Customers are notified of product packaging changes in advance	**4.26**	**3.95**	MBQ	Excellent
31	Batch number and validity period requirements specified by the customer are met	4.01	**3.75**	MBQ	Surplus
32	Customers receive order-processing status online	3.57	3.38	IDQ	Care-free
33	Out-of-stock orders are promptly processed	**4.25**	3.50	MBQ	To be improved
34	Customer complaints are immediately addressed and resolved	**4.21**	**3.73**	MBQ	Excellent
35	Shipment of damaged goods or incorrect invoices and bills of voucher are promptly corrected	**4.33**	**3.86**	MBQ	Excellent
36	Information on materials related to healthcare medicines and procurement advice are provided	3.61	3.37	IDQ	Care-free
37	Services such as medical waste recycling, waste disposal, and autoclaving are available	3.53	3.21	IDQ	Care-free
38	Escrow inventory services are provided	3.24	3.16	IDQ	Care-free

Note: The bold stands for the above average.

**Table 7:** IS analysis of different pharmaceutical logistics service providers.

Items		Professional	General	Both
1	The clothing of delivery staff is neat and tidy	Surplus	Excellent	Surplus
2	Logistics company has good word-of-mouth, reputation, and popularity	Excellent	Excellent	Excellent
3	Logistics center has advanced physical equipment (e.g., warehouses, pickup systems, shelves)	Surplus	Surplus	Surplus
4	Processes including order content (items and quantities) bill of lading documents are correctly executed	Excellent	Excellent	Excellent
5	Processes for batch numbers and validity period management are strictly implemented; i.e., drugs with a validity period of less than 6 months are not shipped	Excellent	To be improved	Excellent
6	The rate and extent of damage received are disclosed/low	Excellent	Excellent	Excellent
7	Logistics equipment and distribution vehicles meet the temperature requirements of drugs	Excellent	Excellent	Excellent
8	Goods are delivered on time to customers	Excellent	Excellent	Excellent
9	The order (i.e., item and quantity) delivery rate is accurate	Excellent	Excellent	Excellent
10	Customer enquiries are answered within the promised period	Excellent	Excellent	To be improved
11	Return and exchange processes are prompt and appropriate	Excellent	Excellent	To be improved
12	Urgent orders are accepted and processed with timely delivery	Excellent	Excellent	Excellent
13	There is a limit on the minimum order amount	Care-free	Care-free	Care-free
14	Customers are notified of product shipment one day prior	Care-free	Care-free	Care-free
15	Single-window customer service staff provides professional and satisfactory answers	Excellent	Care-free	Surplus
16	Deliveries are completed every other day after receiving the order	Excellent	Care-free	Surplus
17	Service personnel (i.e., order and delivery personnel) quickly address delivery errors	Excellent	Excellent	Excellent
18	Customers are notified of delayed shipment	To be improved	To be improved	To be improved
19	Service personnel (i.e., order and delivery personnel) have professional training and certain degree of understanding about drugs	Care-free	Care-free	Care-free
20	Service staff (i.e., order and delivery staff) are kind and courteous	Excellent	Excellent	Excellent
21	Customized logistics processing and packaging services are available	Care-free	Care-free	Care-free
22	Deliveries are made as per time specified by customers	Surplus	Surplus	Excellent
23	Customers are notified of out-of-stock products	To be improved	To be improved	To be improved
24	There is no limit on order time	Care-free	Care-free	Care-free
25	Customers can place online orders using the electronic platform/Online orders are accepted	Care-free	Care-free	Care-free
26	Delivery services are available on weekends and holidays	Care-free	Care-free	Care-free
27	There are channels for customer complaints	Care-free	Care-free	Care-free
28	Inventory location or shelf service is designated as per customer needs	Surplus	To be improved	Surplus
29	Information technology (RFID and barcode) offers information on drug history and temperature control	Care-free	Care-free	Care-free
30	Customers are notified of product packaging changes in advance	Excellent	Excellent	Excellent
31	Batch number and validity period requirements specified by the customer are met	Excellent	Surplus	Care-free
32	Customers receive order-processing status online	Care-free	Care-free	Care-free
33	Out-of-stock orders are promptly processed	To be improved	To be improved	To be improved
34	Customer complaints are immediately addressed and resolved	Excellent	Excellent	To be improved
35	Shipment of damaged goods or incorrect invoices and bills of voucher are promptly corrected	Excellent	Excellent	Excellent
36	Information on materials related to healthcare medicines and procurement advice are provided	Care-free	Care-free	Care-free
37	Services such as medical waste recycling, waste disposal, and autoclaving are available	Care-free	Care-free	Care-free
38	Escrow inventory services are provided	Care-free	Care-free	Care-free
